# EXOSC10/Rrp6 is post-translationally regulated in male germ cells and controls the onset of spermatogenesis

**DOI:** 10.1038/s41598-017-14643-y

**Published:** 2017-11-08

**Authors:** Soazik P. Jamin, Fabrice G. Petit, Christine Kervarrec, Fatima Smagulova, Doris Illner, Harry Scherthan, Michael Primig

**Affiliations:** 1 Inserm U1085 IRSET, Université de Rennes 1, 35000 Rennes, France; 2 Institut für Radiobiologie der Bundeswehr in Verb. mit der Universität Ulm, 80937 Munich, Germany; 3 Present Address: PAN-Biotech, 94501 Aidenbach, Germany

## Abstract

EXOSC10 is a catalytic subunit of the exosome that processes biologically active transcripts, degrades aberrant mRNAs and targets certain long non-coding RNAs (lncRNAs). The yeast orthologue Rrp6 is required for efficient growth and gametogenesis, and becomes unstable during meiosis. However, nothing is known about the localization, stability and function of EXOSC10 in the rodent male germline. We detect the protein in nucleoli and the cytoplasm of mitotic and meiotic germ cells, and find that it transiently associates with the XY body, a structure targeted by meiotic sex chromosome inactivation (MSCI). Finally, EXOSC10 becomes unstable at later stages of gamete development. To determine *Exosc10*’s meiotic function, we inactivated the gene specifically in male germ cells using cre recombinase controlled by *Stra8* or *Ddx4*/*Vasa* promoters. Mutant mice have small testes, show impaired germ cell differentiation and are subfertile. Our results demonstrate that EXOSC10 is post-translationally regulated in germ cells, associate the protein with epigenetic chromosome silencing, and reveal its essential role in germ cell growth and development.

## Introduction

The 3′–5′ exoribonuclease EXOSC10 (also called PM/Scl-100 in human or Rrp6 in yeast and fly) is a catalytic subunit of the multimeric exosome^1–3^. This highly conserved protein complex processes RNA precursors, controls the accuracy of mRNA biogenesis and degrades certain classes of long non-coding RNAs (lncRNAs); for review, see^4^. EXOSC10 also functions without the exosome core subunits, and was recently found to bind RNA alone via a C-terminal domain^5,6^. Interestingly, the protein regulates *Xist* RNA and the onset of X chromosome inactivation in somatic cells^7^. EXOSC10 is of broad clinical importance because it is the target of auto-antibodies produced in patients suffering from polymyositis/scleroderma overlap syndrome (reviewed in^8^), and both yeast and human proteins are directly inhibited by the widely used anti-cancer drug 5-fluorouracil (5-FU)^9,10^. The gene was initially thought to be dispensable for growth in cultured human cells based on siRNA-mediated interference with gene expression^11^. More recent *in vitro* work using cultured cell lines and a gene-trap approach revealed that human *EXOSC10* is essential for mitotic cell division^12^. However, it is not known how the protein is regulated in dividing and differentiating germ cells, and if it is important for mammalian male germline development.

In the male mouse, spermatogonial stem cells respond to external cues by differentiating into A type, intermediate, and eventually B type spermatogonia (reviewed in^13,14^). The latter exit mitotic growth and enter the meiotic developmental pathway that includes two divisions (meiosis I and II) lacking an intervening round of DNA replication. During the process, primary spermatocytes transit through a prolonged prophase I that includes DNA replication and six cytologically distinguishable sub-stages (pre-leptotene, leptotene, zygotene, pachytene, diplotene and diakinesis) during which cells replicate DNA, form the synaptonemal complex (SC) between homologous chromosomes (bivalents), and undergo meiotic recombination^15^. As opposed to autosomes, X and Y chromosomes remain unsynapsed during the pachytene stage, apart from the small pseudo-autosomal region, leading to the formation of the XY- or sex body. Incomplete synapsis is the reason why X and Y chromosomes are transcriptionally silenced *via* an epigenetic process called meiotic sex chromosome inactivation (MSCI) that is critical for male fertility^16,17^. The regulatory mechanisms governing MSCI are not entirely understood but they are thought to be different from *Xist*-mediated X chromosome silencing in females^18,19^. After separation of homologous chromosomes in meiosis I, each secondary spermatocyte separates sister chromatids in meiosis II to generate four round spermatids that carry haploid sets of chromosomes. These post-meiotic cells further differentiate into elongated spermatids and ultimately into spermatozoa that undergo final maturation steps during their passage through the epididymis, which is critical for their ability to fertilize the oocyte (for review, see^20,21^).

Human *EXOSC10* was originally identified using a viral expression library screened with anti-nucleolar antibodies from patients diagnosed with scleroderma-polymyositis overlap syndrome^22,23^. Later, yeast *RRP6* was cloned and the protein was detected in the nucleus^24,25^. In earlier studies, we showed that yeast Rrp6 is important for growth as well as efficient meiosis and gametogenesis (sporulation) in various strain backgrounds, and that the protein becomes unstable as cells progress through the meiotic pathway^26^. These results prompted us to ask if the mammalian *RRP6* homolog *Exosc10* is similarly regulated at the protein level, and if it is required for cell growth and differentiation *in vivo*. We find that EXOSC10 dynamically localizes to nucleoli and adjacent to the XY body in meiotic prophase I and in the cytoplasm during metaphase I, before it concentrates again in the nucleus of secondary spermatocytes and haploid round spermatids. Finally, the protein becomes unstable when post-meiotic germ cells start differentiating into sperm. To study the gene’s function, we have generated an *Exosc10* mutant mouse line using embryonic stem cells provided by the Knock-Out Mouse Project (KOMP; www.komp.org; ref.^27^), and found no viable *Exosc10* ^−/−^ mice (Petit *et al*., in preparation). We therefore carried out a targeted approach based on the cre-*lox* system^28^, and observed that disrupting *Exosc10* in spermatogonia strongly reduces testis size, impairs germline development and diminishes fertility.

We conclude that EXOSC10 is regulated at the level of protein localization and stability during male germ cell differentiation. Our results associate the protein with epigenetic silencing of unsynapsed chromosomes, and demonstrate its essential function in germ cell proliferation and development.

## Results

### *Exosc10* is regulated post-translationally in the male germline

RNA profiling data obtained with microarrays or RNA-Sequencing available in public databases including Genevestigator (www.genevestigator.com), GermOnline (www.germonline.org) and the Human Protein Atlas (www.proteinatlas.org) show that mammalian *Exosc10* is broadly expressed in most embryonic and adult tissues^29–31^. To more specifically assess the testicular expression pattern of mouse *Exosc10*, we used published RNA-Sequencing data obtained with enriched populations of Sertoli cells, and mitotic, meiotic and post-meiotic germ cells at various stages of differentiation, and epigenetic ChIP-Sequencing data obtained with total testis samples^32,33^. As expected, we observed the epigenetic histone H3 lysine 4 tri-methylation mark (H3K4me3) associated with gene activation, and found homogenous expression signals in all testicular cell types (Fig. S1).

### EXOSC10 transiently localizes to the XY body

**Figure 1:**
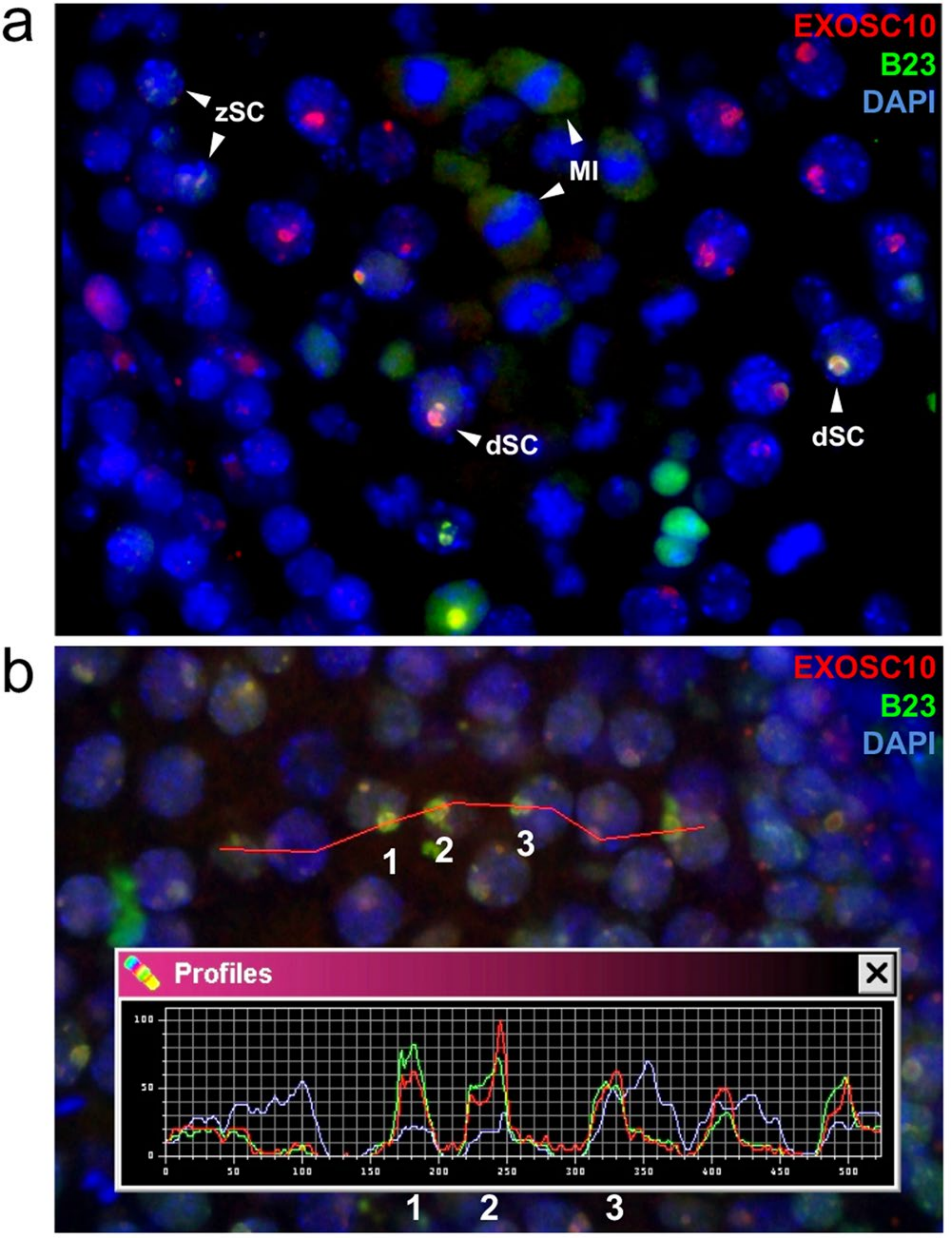
EXOSC10 localization during meiosis I and II in the mouse. (**a**) Immunofluorescence co-staining of EXOSC10 (red) and the nucleolar marker B23 (green) in a stage XII seminiferous tubule. EXOSC10 and B23 nucleolar co-localisation is visualized in orange. Typical cases of zygotene and diplotene spermatocytes (zSC, dSC) and metaphase I (MI) spermatocytes are indicated with white arrowheads. (**b**) Fluorescence profile analysis of EXOSC10 (red) and B23 (green) co-localisation in three large nucleoli of late pachytene spermatocytes. The red line identifies target cells. A color-coded graph quantifies the signal intensities (y-axis) in nuclei identified (x-axis). Three typical nucleoli are numbered.

We monitored EXOSC10 in mouse testis in combination with the B23 nucleolar marker protein and found weak co-staining in the nucleolus of zygotene spermatocytes and strong nucleolar signals during late pachytene and diplotene sub-stages (Fig. 1a,b). Later, EXOSC10 levels decrease and the protein accumulates in the cytoplasm during metaphase I (Fig. 1a,b).

**Figure 2:**
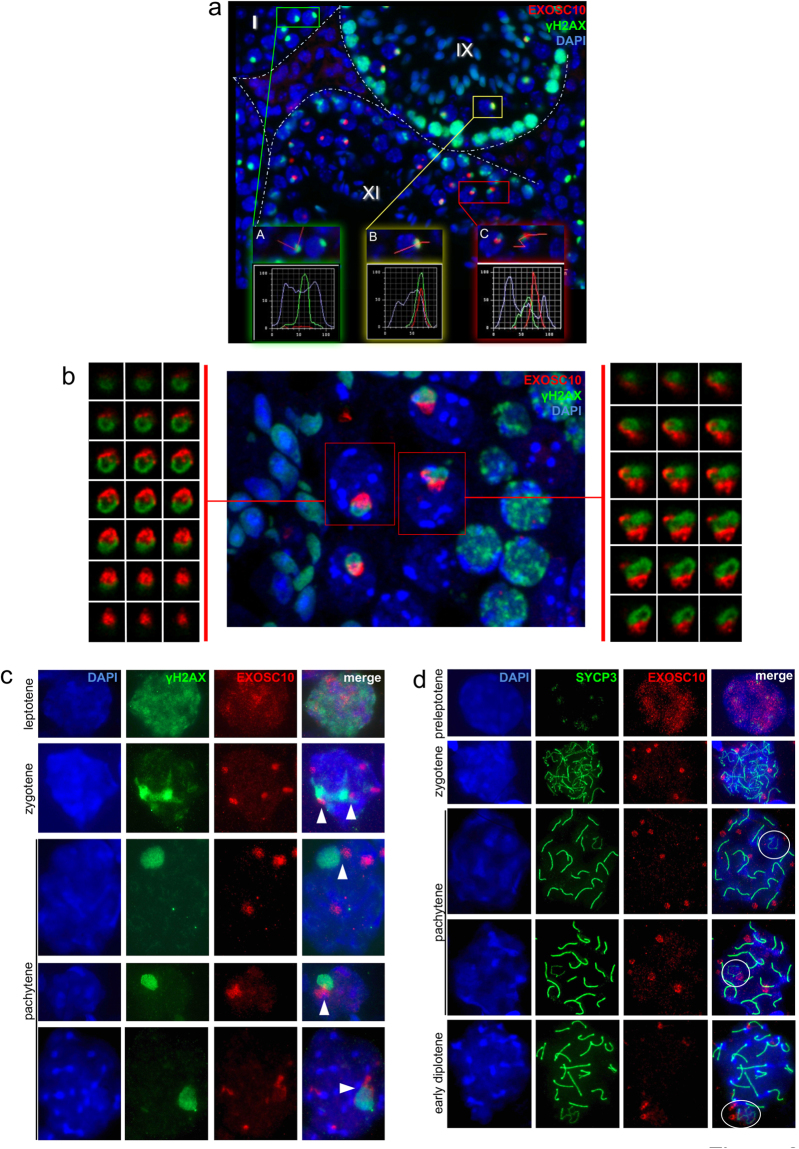
EXOSC10 localization during meiotic germ cell differentiation. (**a**) Dynamic localisation of EXOSC10 (red) during spermatocyte differentiation in comparison to γ-H2AX (green). Fluorescence signal intensities are plotted for XY bodies in early (panel A framed in green), and late pachytene (panel B in yellow), and diplotene (panel C in red). The tubule stage is given. A stippled line outlines the borders of seminiferous tubules of a 35dpp mouse testis. (**b**) Laser scanning microscopic analysis of EXOSC10 (red) and XY body chromatin (γ-H2AX, green) in diplotene XY bodies of a stage XI tubule. The sex bodies of the boxed spermatocytes are shown below as Z-projections of 3 × 1 µm image stacks (from upper left to lower right). (**c**) Immunofluorescence analysis of nuclear spreads at different stages of meiotic prophase I as indicated to the left. Images show DAPI-stained DNA (blue), γ-H2AX (green) and EXOSC10 (red). The rightmost column shows all signals (merge). White arrowheads point to XY bodies associated with an EXOSC10 positive aggregate. (**d**) A similar experiment is shown for prophase I stages that are given to the right, revealing DNA with DAPI (blue), the synaptonemal complex with the SYCP3 marker (green) and EXOSC10 (red). Typical cases of associated XY body-EXOSC10 signals are encircled in the rightmost column (merge).

To further assess the dynamics of EXOSC10 localisation we next co-stained with γ-H2AX, a marker for DNA double strand breaks that specifically labels chromatin during leptotene and zygotene stages and the XY body in pachytene spermatocytes. We found no EXOSC10 protein at the XY body during early pachytene, while in late pachytene both EXOSC10 and γ-H2AX co-localize at the XY body (Fig. 2a, sub-panels A and B in stage I and stage IX tubules). After pachytene, EXOSC10 strongly accumulates adjacent to the diplotene XY body, as revealed in detail by laser scanning microscopy (Fig. 2a, sub-panel C in a stage XI tubule and Fig. 2b). We next monitored γ-H2AX and EXOSC10 localisation patterns during meiotic prophase in nuclear surface spreads. We observe a pan γ-H2AX signal and a diffuse nuclear signal for EXOSC10 during leptonene. In pachytene spermatocytes the γ-H2AX signal is restricted to the XY body, while EXOSC10 aggregates in several sub-structures in the nucleus (that are likely nucleoli; see Fig. 1 ^34^), some of which are associated with the XY body (Fig. 2c). A similar pattern of physical juxtaposition was obtained when nuclear spreads from spermatocytes in pre-leptotene, zygotene, pachytene and early diplotene were analysed for the synaptonemal complex component SYCP3 and EXOSC10 (Fig. 2d). These data are in keeping with an earlier observation that the nucleolus is attached to the XY body during pachytene; reviewed in^34^.

**Figure 3:**
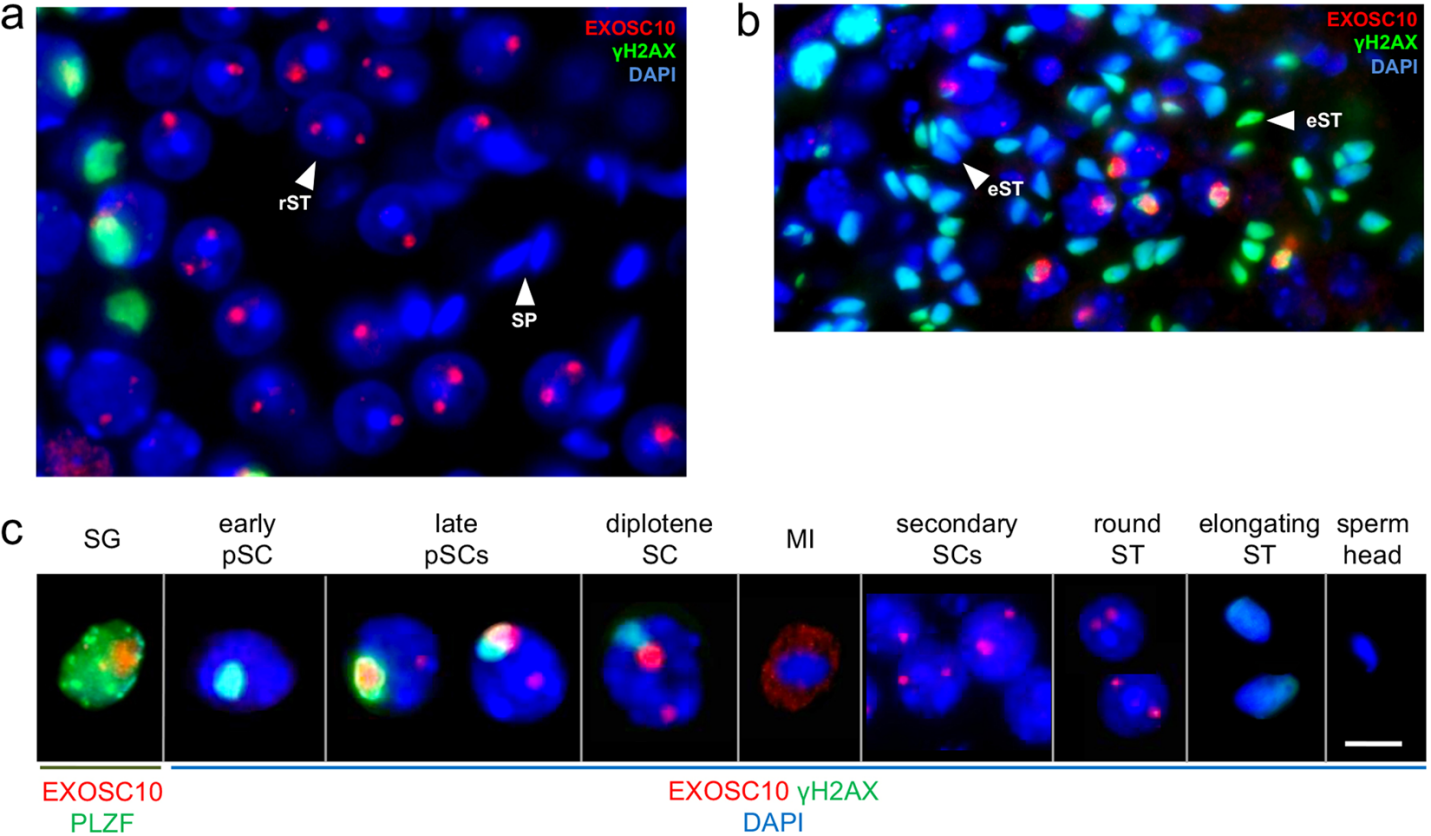
EXOSC10 localization during post-meiotic germ cell differentiation. (**a**) Immunofluorescence co-staining signals for EXOSC10 (red), γ-H2AX (green) and DNA (blue) are shown for round spermatids (rST) and sperm heads (SP) in a stage VI tubule. (**b**) The staining pattern is shown for elongating spermatids (eST) in a stage XI tubule. White arrowheads point to typical examples. (**c**) Typical sequence of EXOSC10 redistribution in spermatogenic nuclei. A PLZF-positive (green) spermatogonium (SG) containing EXOSC10 (red) in the nucleolus is given. EXOSC10 (red) and γ-H2AX-labeled XY body (green) staining is shown for spermatocytes at early pachytene (early pSC), late pachytene (late pSCs), diplotene (diplotene SC), metaphase I (MI), and Meiosis II (secondary SCs), post-meiotic spermatids (round ST, elongating ST) and a sperm head. DNA is stained in all panels with DAPI (blue). Scale bar: 4 µm.

The tendency of EXOSC10 to diminish as cells progress through spermatogenic differentiation is visible when comparing haploid round spermatids to γ-H2AX-positive elongated spermatids undergoing chromatin remodeling: the latter, and mature sperm do not appear to express EXOSC10 (Fig. 3a,b). In summary, we find peak levels of EXOSC10 in nucleoli of PLZF-positive spermatogonia and pachytene/diplotene spermatocytes, and lower levels of EXOSC10 in the nucleoli of zygotene spermatocytes and the cytoplasm of primary spermatocytes undergoing meiosis I. In secondary spermatocytes and post-meiotic round spermatids, we observe residual signals that concentrate in one or two foci. In round spermatids, the nucleolar signal forms dots adjacent to the strongly DAPI-stained chromocenters, which are likely associated with the subcentromeric nucleolus organizing regions (NOR) of the acrocentric mouse chromosomes. No EXOSC10 protein is detectable in elongating spermatids and sperm heads (Fig. 3c).

To further validate our data across mammalian species, we investigated the testicular EXOSC10 staining pattern in histological sections from an adult rat in combination with SYCP3, which marks pachytene spermatocytes. Again, we find EXOSC10 mostly in nucleoli of leptotene, pachytene and diplotene spermatocytes but no signals in elongating spermatids (Fig. S2a,b in stage IX and XIII tubules). In addition to immunofluorescence assays, we also monitored EXOSC10 levels in protein extracts from enriched populations of spermatogonia, spermatocytes and spermatids as compared to total testis by Western blotting, and found that the protein was below the threshold level of detection in the spermatid fraction (Fig. S2c). This finding confirms the tendency of EXOSC10 protein levels to decrease as spermatogenesis progresses.

Our results show that mouse EXOSC10 is mostly concentrated in the nucleolus of mitotic, meiotic and early post-meiotic germ cells, and that the protein accumulates to low levels in the cytoplasm of primary spermatocytes undergoing meiosis I, which indicates that the protein’s localisation is regulated during the cell cycle. We also find that EXOSC10 becomes undetectable as cells exit the second meiotic metaphase and enter spermiogenesis. This pattern is broadly confirmed in the rat, and reminiscent of diminishing yeast Rrp6 protein levels as cells switch from mitosis to meiosis and spore formation. The data are thus consistent with the idea that the post-translational mechanism of EXOSC10/Rrp6’s regulation during gametogenesis is conserved during evolution^26^.

### Spermatogonial *Exosc10* is important for normal testis development

**Figure 4:**
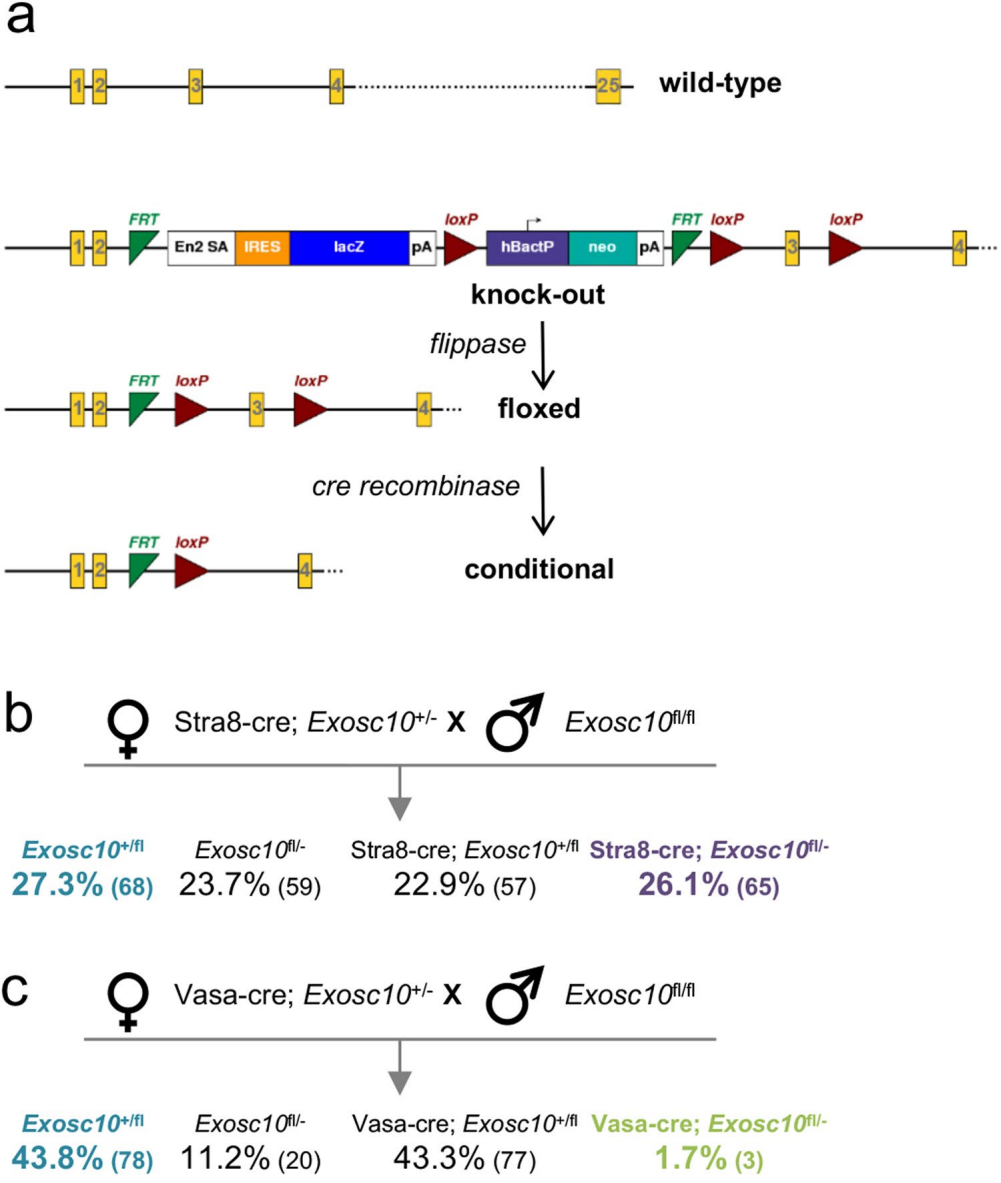
Targeted *Exosc10* gene deletion models. (**a**) A schematic shows the first four and the last out of 25 exons in yellow for wild-type, knock-out (knock-out first, promoter driven; see www.komp.org/alleles.php#nonconditional-promoter-csd), floxed and conditional knock-out (cKO) alleles. Red triangles symbolize cre recombinase cleavage sites (*lox*P) that flank exon 3 to conditionally inactivate *Exosc10*. Green triangles represent the *flippase* recognition target (*FRT*) sites used to remove the *neomycin* selectable marker and the *lac*Z reporter gene. (**b**,**c**) The detailed female and male genotypes of the parents and the F1 offspring is shown for Stra8-cre (**b**) and Vasa-cre (**c**). The percentages of each genotype among the total number of newborn mice is shown and the corresponding numbers are given in round brackets.

To study the function of *Exosc10 in vivo*, we have generated an *Exosc10* knockout mouse line from embryonic stem cells provided by KOMP (www.komp.org; *Exosc10* knockout first, promoter driven; see methods for more detail). We found no *Exosc10* ^−/−^ mice among the offspring (Petit *et al*., in preparation), and therefore we used the cre-*lox* system to target *Exosc10* specifically in the male germline. To generate the *Exosc10* floxed line (*Exosc10* ^fl^), the *Exosc10* knockout first line was crossed with the Gt(ROSA)26Sor^tm2(CAG-flp^°^,-EYFP)lcs^ mouse line at the Mouse Clinical Institute (ICS, Strasbourg, France)^35^. In the *Exosc10* ^fl^ mouse the third exon is flanked by two *lox*P sites (Fig. 4a). We used the well-established STOCK Tg(Stra8-icre)1Reb/J line (refered to as Stra8-cre) that expresses the cre recombinase in spermatogonia and pre-leptotene spermatocytes. In addition, we used FVB-Tg(Ddx4-cre)1Dcas/J mice (refered to as Vasa-cre) that initiate cre expression in early undifferentiated spermatogonia^36,37^. Since *Exosc10* ^fl^ mouse line is in a C57BL/6 genetic background, we have backcrossed our Stra8-cre and Vasa-cre mouse lines with the C57BL/6NRj mouse strain (Janvier, Le Genest Saint Isle, France) at least five times before generating *Exosc10* conditional knockout mouse lines.

To optimize the inactivation of *Exosc10*, the *Exosc10*-null and fl alleles were combined. We generated Stra8-cre; *Exosc10* ^+/−^ female mice, which were crossed with *Exosc10* ^fl/fl^ males. As shown in Fig. 4b, the Stra8-cre; *Exosc10* ^fl/−^ (referred to as *Exosc10* ^cKO(Stra8)^) were obtained at the expected Mendelian frequency. However, the outcome was different when we crossed Vasa-cre; *Exosc10* ^+/−^ female mice with *Exosc10* ^fl/fl^ male mice (Fig. 4c). Vasa-cre; *Exosc10* ^fl/−^ (referred to as *Exosc10* ^cKO(Vasa)^) were obtained at the frequency of only 1.7%. Ectopic expression of Vasa-cre and the possible presence of cre in the egg could lead to embryonic lethality as observed in *Exosc10*-null mice, and therefore may explain this unexpectedly low frequency^36^. Furthermore, the frequency of *Exosc10* ^fl/−^ mice is smaller than expected (11.2%), which is likely due to persisting cre protein from the egg^36^.

**Figure 5:**
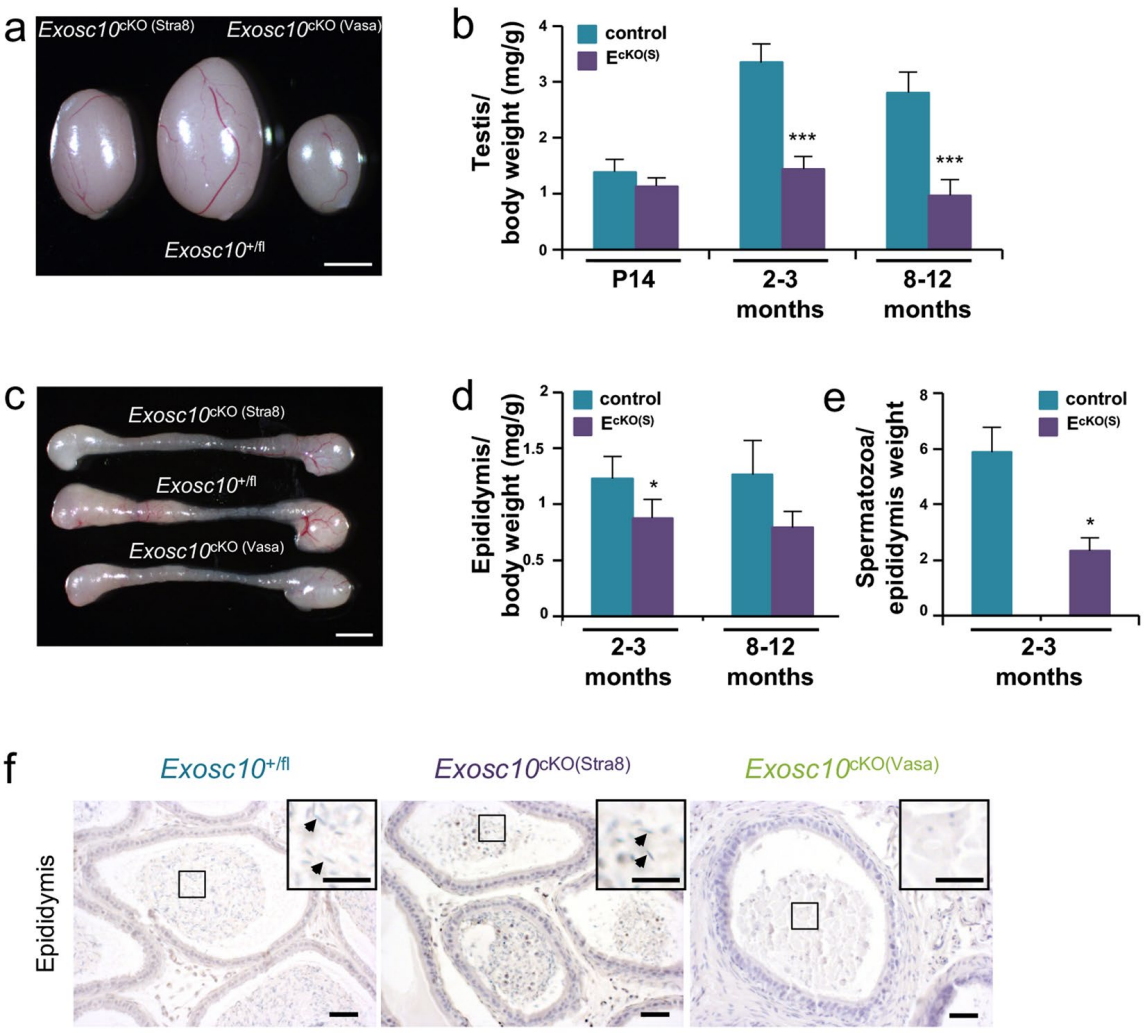
Morphology of testis and epididymis in targeted deletion mutants. (**a**) Testes from 8-week-old control and targeted mutant mice (*Exosc10* ^cKO(Stra8)^ and *Exosc10* ^cKO(Vasa)^) are shown. Scale bar: 2 mm. (**b**) A color-coded graph plots the weight of testis over body-weight (y-axis) for controls (C in blue) and Stra8-cre mutants (*Exosc10* ^cKO(Stra8)^ in purple) at the ages indicated at the bottom (x-axis). (P14: control, n = 6; mutant, n = 4; p = 0.171/2–3 months: control, n = 7; mutant, n = 11/8–12 months: control, n = 10; mutant, n = 12; ***p < 0.001, non parametric Mann-Whitney test). The data are presented as median values ± SD. (**c**) Epididymides from 8-week-old control and mutant mice are shown. Scale bar: 2 mm. (**d**) A color-coded graph plots the weight of epididymides over the body weight. (2–3 months: control, n = 6; mutant, n = 8; *p < 0.05/8–12 months: control, n = 7; mutant, n = 6; p = 0.0734, non parametric Mann-Whitney test). The data are presented as median values ± SD. (**e**) The number of spermatozoa over the weight of epididymis is plotted. (control, n = 3; mutant, n = 4, *p < 0.05, non parametric one-tailed Mann-Whitney test). The data are presented as median values ± SD. (**f**) Hematoxylin/eosin staining of paraffin-embedded epidydimis tails from control, *Exosc10* ^cKO(Stra8)^ and *Exosc10* ^cKO(Vasa)^. Black arrowheads point to spermatozoa. Scale bar: 20 μm.

To characterize the *Exosc10* ^cKO(Stra8)^ phenotypes, we isolated the gonads of two-month-old mutant and control mice, and found that mutant testes were smaller than those of the controls. An even more pronounced effect was visible in the case of *Exosc10* ^cKO(Vasa)^ mutant mice (Fig. 5a). We then compared the average weight of *Exosc10* ^cKO(Stra8)^ and control testes at 14 days post-partum (dpp), 2–3 months and 8–12 months, and observed that testis weight diminished by about 60% in mature *Exosc10* ^cKO(Stra8)^ mice (57% at 2–3 months and 65% at 8–12 months, Fig. 5b). The testis/body weight ratio of *Exosc10* ^cKO(Vasa)^ mice was 80% lower than the control (Fig. S3, 0.670 ± 0.010 mg/g (n = 3) versus 3.347 ± 1.127 mg/g (n = 7) for the control; p = 0.0167). Moreover, while the control testis weight increases by 2.7 fold between 2 weeks and 2 months, the mutant testis does not develop significantly (Fig. 5b). For the epididymis, it appears that the length and shape are similar between control and mutant mice. However, *Exosc10* ^cKO(Stra8)^ and *Exosc10* ^cKO(Vasa)^ epididymis appear less opaque than the control (Fig. 5c), and the epididymis/body weight ratio is significantly reduced (Fig. 5d for *Exosc10* ^cKO(Stra8)^). A similar result is observed for *Exosc10* ^cKO(Vasa)^ (Fig. S3, 0.700 ± 0.005 mg/g (n = 3) versus 1.228 ± 0.081 mg/g (n = 6) for the control; p = 0.0238). The epididymis sperm count was significantly lower at 2–3 months in *Exosc10* ^cKO(Stra8)^ mice (Fig. 5e). A histological analysis of the caudal epididymis revealed that in *Exosc10* ^cKO(Stra8)^ males the number of sperm is reduced as compared to the control. In *Exosc10* ^cKO(Vasa)^, the inactivation of *Exosc10* leads to an even more dramatic phenotype since no sperm is observed in the lumen of the tubules (Fig. 5f). This observation suggests that spermatogenesis is strongly affected in mutant male mice and, consequently, fertility should be reduced.

Therefore, we next compared the reproductive capacity of control and *Exosc10* ^cKO(Stra8)^ male mice by mating them with wild-type (wt) females (Table 1). For this analysis, males were used between 2- and 12-months of age, and were mated with two wt female mice at a time (females were mated between 2- and 6-months of age). The results showed that only 45.5% of female mice that mated with *Exosc10* ^cKO(Stra8)^ males gave birth, producing 97 offspring in 18 litters, while 96% of the females mated with control males gave birth to 580 pups in 78 litters. The average litter size is 5.4 in the *Exosc10* ^cKO(Stra8)^ background versus 7.6 in the control (Table 1). These data are in agreement with the low sperm count and show that *Exosc10* ^cKO(Stra8)^ male mice are hypofertile.

**Table 1:** Reproductive capacity of control *versus* mutant mice.

		Control	Mutant
**Males**	**2**–**12 mo**	**6**	**4**
	2–6 mo	6	4
	6–12 mo	7	7
**WT females**	**2**–**12 mo**	**25**	**22**
	2–6 mo	15	9
	6–12 mo	18	21
**WT females w/pups**	**2**–**12 mo**	**24 (96%)**	**10 (45.5%)**
	2–6 mo	14 (93.3%)	6 (66.7%)
	6–12 mo	18 (100%)	4 (19%)
**Pups**	**2**–**12 mo**	**580**	**97**
	2–6 mo	268	58
	6–12 mo	357	39
**Litters**	**2**–**12 mo**	**78**	**18**
	2–6 mo	37	12
	6–12 mo	47	6
**Litter size**	**2**–**12 mo**	**7.6 ± 2.4**	5.4 ± 3.4**
	2–6 mo	7.2 ± 2.4	4.8 ± 3.5*
	6–12 mo	7.8 ± 2.6	6.5 ± 3.1

Since the inactivation of *Exosc10* by Stra8-cre might become more efficient over time, we analyzed the reproductive capacity of male mice at 2–6 versus 6–12 months. The results showed that although the litter size was slightly reduced in the case of *Exosc10* ^cKO(Stra8)^ male mice as compared to control mice (4.8 ± 3.5 versus 7.2 ± 2.4 for 2–6 months (p = 0.0103) and 6.5 ± 3.1 versus 7.8 ± 2.6 for 6–12 months (p = 0.2222), Table 1), no significant difference was observed between younger and older mice. However, we note that only 19% of wild type females  were fertilized by 6–12-month-old males versus 66.7% in the case of 2–6-month-old males (Table 1), while 2–6- and 6–12-month-old control mice were able to fertilize more than 93% of wild type females. We conclude that *Exosc10* ^cKO(Stra8)^ male mice are less capable than control mice to fertilize females, and their reproductive capacity further diminishes with increasing age. However, the efficiency of fertilization by sperm from mutant mice is not affected by the lower quantity of sperm as shown by the litter size.

Due to a very low number of *Exosc10* ^cKO(Vasa)^ males, we were unable to assess the reproductive capacity of this line. However, since the epidydimis’ lumen is devoid of sperm, we conclude that *Exosc10* ^cKO(Vasa)^ male mice are most likely infertile.

Taken together, our results show that targeted inactivation of *Exosc10* in male germ cells significantly reduces the weight of testis and diminishes the number of sperm that are produced. The data also demonstrate that, in the absence of *Exosc10*, spermatogenesis is severely impaired, albeit not completely abolished. This result can be explained in two ways. Either, contrary to mitosis, *Exosc10* is not essential for the meiotic cell cycle because another ribonuclease fulfils a partially redundant function in the germline. Alternatively, it is possible that Stra8-driven cre recombinase inactivates *Exosc10* only in a subpopulation of spermatogonia that arrests during cell division and as a consequence is rapidly eliminated from the testis, while the remaining spermatogonia continue to differentiate into sperm^38,39^. The phenomenon of incomplete penetrance is well known; for review, see^28^. To further investigate these possibilities, we histologically examined testicular tissue, and monitored germ cell marker expression in *Exosc10* ^cKO(Stra8)^ and *Exosc10* ^cKO(Vasa)^ mice.

### Male germline development is partially disrupted in *Exosc10*^cKO(Stra8)^ mice and severely impaired in the *Exosc10*^cKO(Vasa)^ line

**Figure 6:**
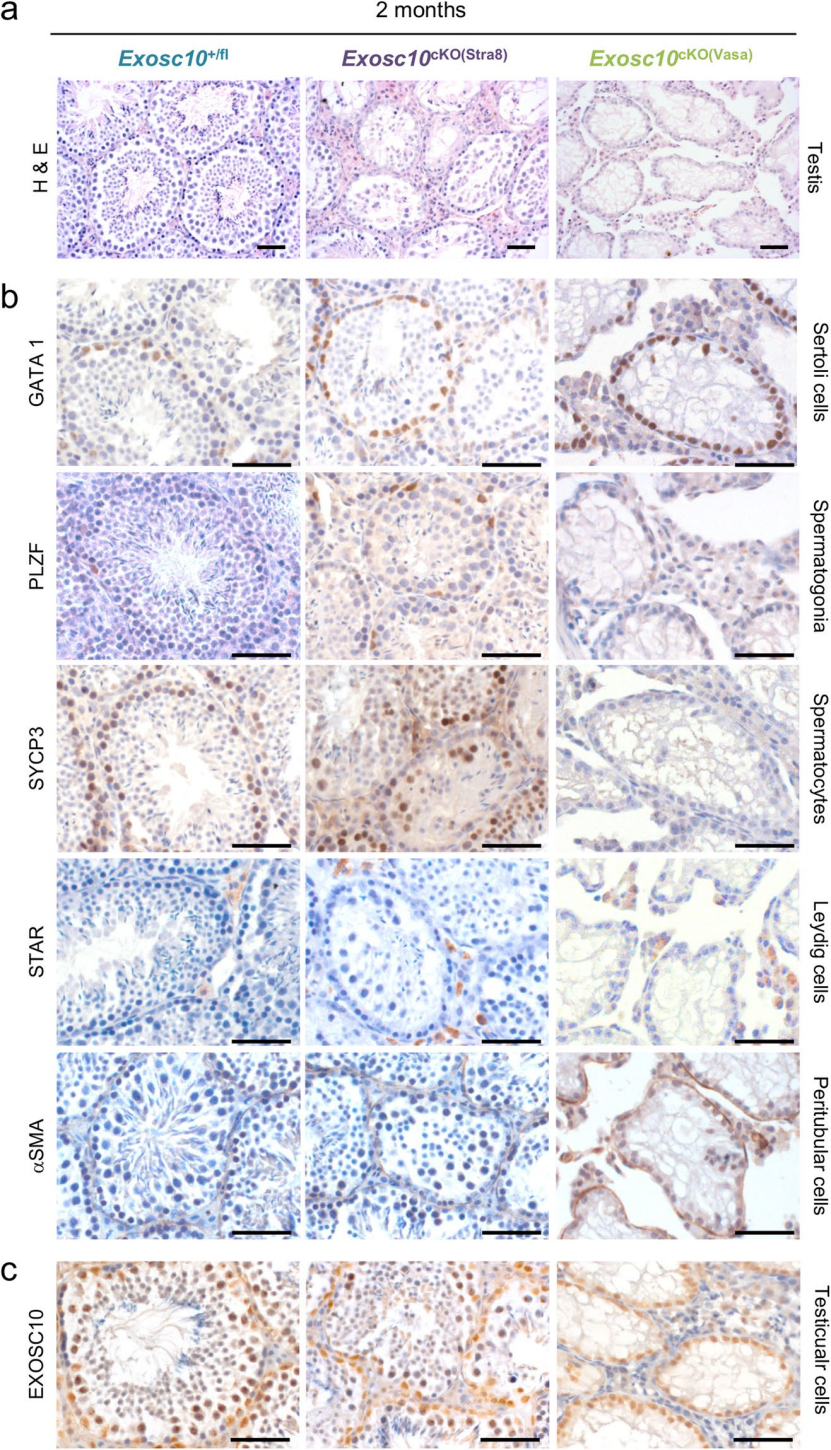
Histology and marker gene expression in epididymis and testis of targeted deletion strains at two months. (**a**) Testicular and epididymal sections stained with hematoxylin/eosin (H&E) are shown with a color code for control (*Exosc10* ^+/fl^ in blue), *Exosc10* ^cKO(Stra8)^ mutant (in purple) and *Exosc10* ^cKO(Vasa)^ mutant (in green) samples as given at the top. Tissue types are indicated to the right. (**b**) Testicular sections examined by immunohistochemistry are shown. Marker proteins are indicated to the left, cell types to the right. (**c**) Testicular sections assayed for EXOSC10 are given like in panel b. Scale bar: 50 μm.

We first analysed the testicular histology of two-month-old control and targeted mutant mice using Bouin-fixed and paraffin-embedded sections to preserve the architecture and integrity of seminiferous tubules, and sections that were stained with hematoxylin/eosin. The overall organization of testes from *Exosc10* ^cKO(Stra8)^ appears to be normal but the seminiferous tubules are smaller than in the control testes (Fig. 6a). Note that the effect of targeted *Exosc10* inactivation in *Exosc10* ^cKO(Stra8)^ on the architecture of the seminiferous tubules is not homogenous, since normal and more or less disorganized tubules are present on the same section. Some tubules show an almost complete exfoliation of germ cells from the epithelium to the lumen (Fig. 6a). In the case of *Exosc10* ^cKO(Vasa)^ testes, the phenotype was even more dramatic: the seminiferous tubules are lined by a single layer of Sertoli cells and utterly depleted of germ cells. As a consequence, the tubules look shrunk and shapeless leaving empty spaces between the tubules and the Leydig cell islets (Fig. 6a).

To analyse the spermatogenesis defect in our mutant mice, we then monitored by immunohistochemistry (IHC) the expression of cell markers in paraformaldehyde-fixed and paraffin-embedded testes from two-month-old mice. Marker proteins for Sertoli cells (GATA1), interstitial Leydig cells (STAR) and peritubular myoid cells that surround the seminiferous tubules (αSMA) were detectable in all samples. This indicates that cells supporting spermatogenesis and tubule architecture are not visibly affected by the absence of EXOSC10 in the germline (Fig. 6b). As opposed to that, marker proteins for undifferentiated spermatogonia (PLZF) and primary spermatocytes (SYCP3) were detected in control and *Exosc10* ^cKO(Stra8)^ testis samples but not in *Exosc10* ^cKO(Vasa)^ tissues (Fig. 6b). In seminiferous tubules from *Exosc10* ^cKO(Stra8)^ mice, fewer spermatocytes were stained than in the control. This is in agreement with the reduced number of spermatids and the hypofertility phenotype of this line. In *Exosc10* ^cKO(Vasa)^ mouse testes, germ cells appear to be completely absent, and we observe numerous vacuoles in the seminiferous tubules that likely occupy space left by missing germ cells (Fig. 6b)^40^.

If our assumption that spermatogonia harboring cre-inactivated *Exosc10* alleles cannot divide and thus fail to enter into the spermatogenic pathway is correct, the remaining germ cell population should express EXOSC10. We indeed detected the protein in residual spermatogonia, spermatocytes and round spermatids present in *Exosc10* ^cKO(Stra8)^ mutant tubules, while no signal is detected in the *Exosc10* ^cKO(Vasa)^ line. These observations confirm that Stra8-driven cre recombinase is not active in all spermatogonia and therefore yields only a partial targeted deletion phenotype. Finally, we confirmed that Sertoli cell nuclei (which do not express Stra8-cre) contain EXOSC10 in both *Exosc10* ^cKO(Stra8)^ and *Exosc10* ^cKO(Vasa)^ mice (Fig. 6c).

**Figure 7:**
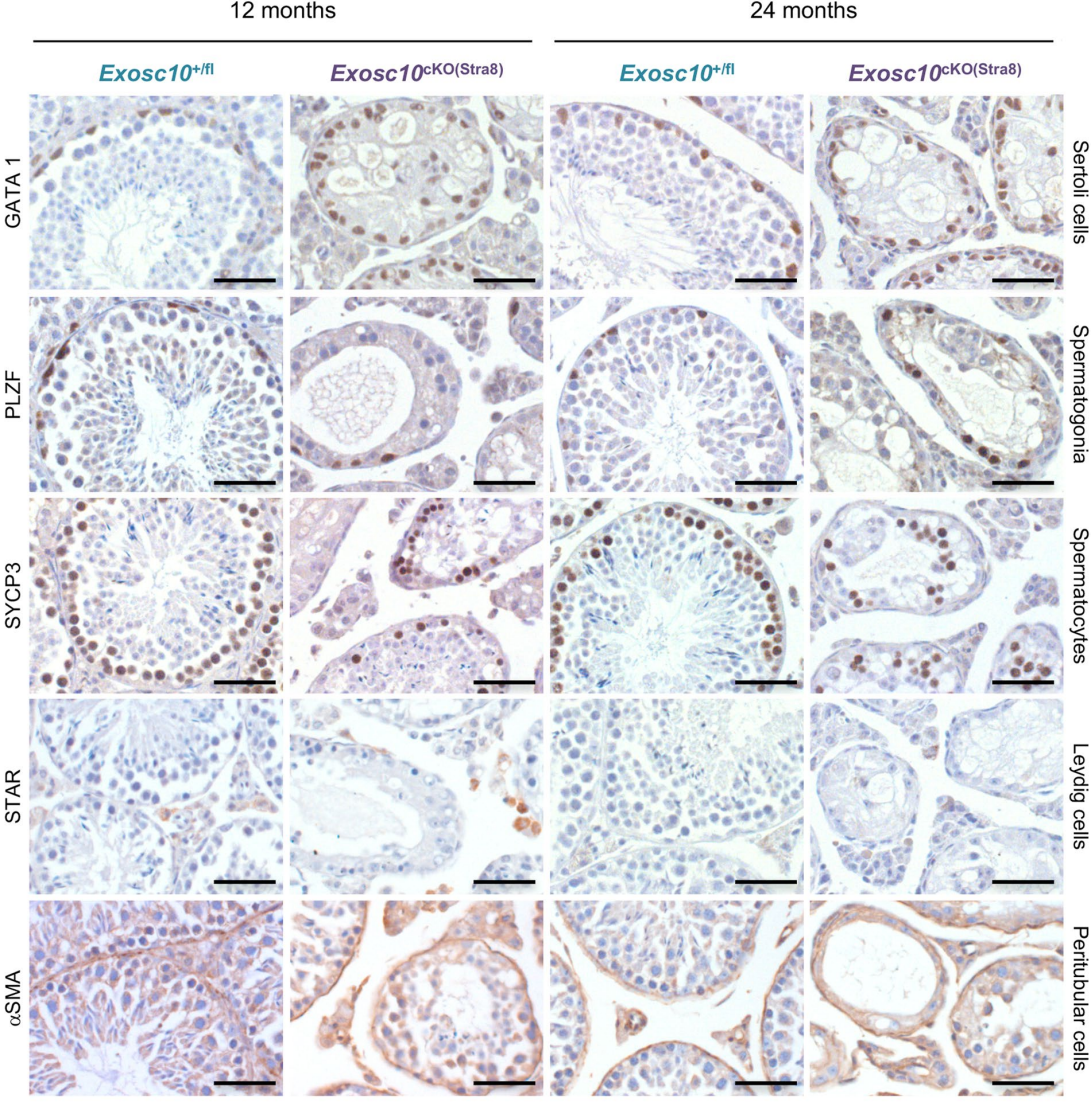
Marker gene expression in targeted deletion strains at 12 and 24 months. Testicular sections from control and mutant mice are shown like in Fig. 6. Samples were taken from adult mice at two different ages as indicated at the top. Scale bar: 50 μm.

Given the incomplete penetrance of cre activity in two-month-old *Exosc10* ^cKO(Stra8)^ mice (Fig. 6b) and the suboptimal capacity of the mutant males to fertilize wild type females (Table 1), we next asked if the histological phenotype was more pronounced in adult mice at 12 and 24 months of age. Using testicular markers for spermatogonia, as well as Sertoli-, Leydig-, and peritubular myoid cells, we observed no significant difference between mutants and controls at 12 and 24 months (Fig. 7). However, the tubular architecture was increasingly damaged in the oldest mice and the number of spermatocytes and spermatids strongly decreased (Fig. 7). Diminishing numbers of germ cells are accompanied by an increasing number of vacuoles and the remaining spermatocytes are largely disorganized (Fig. 7, see panels showing SYCP3 immuno-labelling).

Taken together, our anatomical, histological and immunohistochemical analyses in *Exosc10* ^cKO(Stra8)^ and *Exosc10* ^cKO(Vasa)^ mouse lines indicate that *Exosc10* is required for normal testis size and mitotic division of spermatogonia. This result is in agreement with the human gene’s mitotic function in cultured cells^12^.

## Discussion

We find that mouse EXOSC10 is present at variable levels in nucleoli, the XY body and the cytoplasm of mitotic, meiotic and post-meiotic germ cells, before it becomes undetectable when cells exit meiosis and enter spermiogenesis. This pattern points to multiple cellular sites of action and possibly a regulatory mechanism involving targeted destruction in the male germline. Human EXOSC10 accumulates in B23/nucleolin-positive nucleoli and so-called rarefaction zones of type A dark (Ad) spermatogonia but is undetectable in spermatogonia that are positive for Ki-67, which is a marker for rapidly proliferating spermatogonial cells^41^. This indicates that mammalian EXOSC10 is regulated at the post-translational level via localisation and, depending on whether spermatogonia undergo slow or rapid mitotic cell cycles, protein stability.

We find that rodent EXOSC10 is controlled at the level of protein localization and turnover in the meiotic differentiation pathway, in a way reminiscent of what we have observed for yeast Rrp6 in earlier work^26^. These results may indicate that the mechanism governing Rrp6 and EXOSC10 protein stability during gametogenesis is conserved, although its precise nature remains unknown. We can only speculate what causes the protein to become unstable, but it is perhaps relevant to note that EXOSC10 is ubiquitinated, and therefore likely a target of the Anaphase Promoting Complex/Cyclosome (APC/C). The APC/C attaches ubiquitin to proteins that contain target motifs (degrons), thereby tagging them for degradation by the proteasome, which is critical for both mitosis and meiosis^42^. Given that the complex is active during gametogenesis it is conceivable that it triggers EXOSC10’s degradation during the process; for review, see^43–45^.

Nucleolar localisation of mouse EXOSC10 in meiotic germ cells is consistent with previous observations in human mitotic spermatogonia^46^. We also found that the protein was transiently associated with the XY body in pachytene spermatocytes. It is unclear which mechanism controls this phenomenon but XY body-nucleolus interactions could play a role^47^. Given that EXOSC10 controls various mammalian lncRNAs it is conceivable that the protein’s proximity to the XY body reflects a direct or indirect role for it, possibly *via* its lncRNA targets, in helping to establish or maintain MSCI^48^. This idea is also broadly consistent with EXOSC10’s role in *Xist*-mediated somatic X-chromosome inactivation^7^.

Why does EXOSC10 become unstable during later stages of meiotic development? Transcription of RNAs and translation of proteins must still be able to occur for some time, while germ cells wind down RNA/protein synthesis when they compact their chromatin prior to packaging it into an inert sperm head. It is not entirely understood how ribosome biogenesis and activity are modified in post-meiotic germ cells, but it is known that the eukaryotic proteome diminishes as cells progress through gametogenesis; see^49^ and references therein. It was also found that the ratio of 28S/18S rRNA is altered in human spermatozoa, indicating that ribosome assembly is impaired; this alteration is likely due to extensive rRNA cleavage during spermiogenesis^50,51^. This is interesting in light of Rrp6’s essential role for processing 5.8S rRNA in yeast^24^. It is also noteworthy that stress-induced down-regulation of EXOSC10 in a cultured cell system negatively affects ribosome biosynthesis, and that the protein interacts with MPHOSPH6, an enzyme associated with the exosome that is involved in processing 7S precursor rRNA into mature 5.8S rRNA^52–54^. Taken together, the results support the notion that protein translation in male germline development might at least in part be down-regulated via progressive depletion of EXOSC10.

We initially observed that *Exosc10* is important for early embryogenesis in a gene deletion study (Petit *et al*., in preparation). We therefore used a targeted gene deletion approach based on the cre-*lox* method to study the gene’s role in the male germline, and found that *Exosc10* is also non-redundant and essential for spermatogonial proliferation in the mouse. Our physiological and histological analyses of mutant mice showed partial spermatogenesis and hypofertility in *Exosc10* ^cKO(Stra8)^ mice mediated by residual EXOSC10-positive spermatogonia. The hypomorphic Stra8-cre allele causing incomplete target inactivation is the likeliest explanation for this phenotype. As opposed to that, germ cell populations were entirely depleted in the *Exosc10* ^cKO(Vasa)^ line. However, since Vasa-cre activity is induced between E15 and E18 and occurs in very early undifferentiated spermatogonia^36^, it is not surprising that germ cells are absent in this line.

These results are consistent with an essential role for *Exosc10* during the mitotic division of spermatogonia. It is currently unclear what that critical function might be, since it is not feasible to obtain *Exosc10*-mutant germ cell populations for further analysis by RNA profiling. This technical issue notwithstanding, our work clarifies the important question if *Exosc10* is essential for mouse male germ cell division. This was not certain, since yeast *RRP6* is annotated as a non-essential gene, and human *EXOSC10* was reported to be dispensable for cell growth and division^11,24,26,55^. The testicular data reported here are thus in agreement with our embryological results (Petit *et al*., in preparation) and recent work showing an essential role for human *EXOSC10* in the haploid KBM7 cell line^12^.

It is remarkable that 5-FU was shown to be among the chemotherapeutical compounds that have the strongest cytotoxic effect on the rodent male germline. More specifically, the drug was found to substantially deplete the spermatogonial stem cell population in testicular tissue^56^. Our results, revealing an essential role for *Exosc10* in the proliferation of spermatogonia, together with previous work showing that 5-FU directly inhibits human EXOSC10, highlight a plausible molecular mechanism for this clinically relevant observation^10^.

It is currently unclear if the phenotype we found is exclusively caused by EXOSC10’s exosome-associated nuclear exoribonuclease activity, or if other molecular functions related to exosome-independent DNA/chromatin/RNA-binding also play a role. In this context, it will be an interesting challenge to find out if non-lethal *EXOSC10* alleles exist in humans that specifically affect the male germline.

## Methods

### Published RNA-profiling data display

To visualize testicular *Exosc10* expression, we used published RNA-Sequencing data obtained with highly enriched testicular cells including Sertoli cells, primary spermatogonia, A and B type spermatogonia, leptotene and pachytene spermatocytes, and round and elongated spermatids, in combination with epigenetic profiling data for the H3K4me3 activation mark^32,33^. Data files in tdf format were imported into the Integrated Genomics Viewer (IGV) version 2.3.90, and displayed at log2 scale at a data range of 0–400^57^.

### Ethics Statement

Studies involving animals, including housing and care, method of euthanasia, and experimental protocols, were conducted in accordance with the French regulation for Laboratory Animal Care, issued from the transposition of the European Directive 2010/63/UE. This Directive stipulates in article 1, alinea 2 that euthanizing laboratory animals for the sole purpose of investigating their tissues does not require prior authorization by an ethics committee. The animal facility was licensed by the French Ministry of Agriculture (C35-238-19). Animals were sacrificed using CO2, according to a method in annex IV of the directive. All experiments were supervised by Soazik P. Jamin and Fabrice G. Petit who are licensed for animal experimentation by the French Ministry of Agriculture (92–299 and A92–313, respectively).

### Immunofluorescence analysis of EXOSC10 localization

Testes from mice in the B6C3F1 background (MPI, Berlin, Germany) were fixed over night in neutral buffered 4% formaldehyde (Carl Roth, Karlsruhe, GER), washed 3 × 5 min in PBS/0.1% Glycin (Carl Roth), dehydrated in a graded Ethanol series and embedded in Paraffin (Carl Roth) according to routine methods. Immunostaining of paraffin embedded mouse testis tissue sections was done as described using citrate buffer pre-treatment^58^. Briefly, the sections were first dewaxed and then treated for 1 h in 50 mM sodium citrate at 94 °C. Sections were allowed to cool down to room temperature before they were washed and analyzed using a polyclonal antibody against EXOSC10 (Abcam ab50558; 1:100), and monoclonal antibodies against B23 (Sigma, B0556; 1:500) and γH2AX (Merck Millipore, JBW301; 1:250). Signals were revealed with donkey anti-rabbit Cy3 Fab (Dianova, 711-167-003; 1:1000) and goat anti-mouse Alexa 488 (Mobitec, A11017; 1:500) secondary antibodies^59^.

Images of immuno-stained mouse cells were recorded using the ISIS image analysis system (MetaSystems, Altlussheim, Germany). In some cases, 3D image stacks (step size 0.4 µm or 1 µm) were converted to maximum projection images. Fluorescence profile analysis was done using the measurement option in the ISIS imaging software (MetaSystems). Confocal laser scanning imaging and collection of focus stacks was performed on a TCS SP5 confocal laser-scanning microscope equipped with a Plan Apo 63x/1.4 NA oil immersion objective (voxel size 50 × 50 × 200 nm; Leica) and lasers with excitation lines at 405, 488, 561, 594 and 633 nm.

Immunofluorescence analyses in adult Sprague Dawley rats (Janvier, Le Genest Saint Isle, France) were done using a standard protocol that involves paraformaldehyde perfusion to fix testicular tissue. Samples were treated with 50 mM sodium citrate at pH 6 and saturated with 1x PBS and 2% BSA. Primary antibodies were applied over night at 4 °C. Slides were washed three times in 1x PBS and 0.1% TWEEN before the secondary antibody was added and incubated at room temperature for one hour. We used a polyclonal antibody against EXOSC10 (Abcam, Ab50558; 1:600), and a monoclonal antibody against SYCP3 (Abcam, Ab97672; 1:200). To reveal the signals, we employed chicken anti-rabbit (Alexa, 594) and goat anti-mouse (Alexa, 488) secondary antibodies diluted at 1:500. The samples were analysed using an AxioImager microscope equipped with an AxioCam MRc5 camera and AxioVision software v. 4.8.2 (Zeiss, Le Pecq, France).

### EXOSC10 localization in meiotic surface spreads

The surface spreads were prepared as described in a previous study using testicular samples from 12-week old adult C57BL/6jRj male mice^60^. The spreads were co-immunostained for phosphorylated histone H2AX (**γ**H2AX; green, 05-636, Millipore), and SYCP3, a protein in the axial element of the synaptonemal complex (red, sc-74569, Santa Cruz) or EXOSC10 (red, ab50558, Abcam). The images were taken using an AxioImager microscope equipped with an AxioCam MRc5 camera and AxioVision software version v4.8.1 (Zeiss, Le Pecq, France).

### Western blotting

For Western blot analysis, we collected enriched mitotic (spermatogonia, SG), meiotic (spermatocytes, SC) and post-meiotic (spermatids, ST) germ cell populations and total testis (TT) samples from adult Sprague Dawley rats, and prepared protein extracts using standard protocols as published^61–63^. 20 μg of total protein extract was loaded on a mini-PROTEAN TGX 4–20% precast gel (Bio-Rad) and transferred on PVDF membranes (Merck Millipore, ISEQ. 00010). The membranes are incubated with a rabbit polyclonal anti-EXOSC10 antibody (Abcam, ab50558; 1:800) overnight at 4 °C. After three washing steps, an HRP-conjugated goat anti-rabbit antibody was incubated for 1 h. Signals were revealed with an Amersham ECL Prime kit (GE Healthcare, RPN2232). An anti-β-tubulin antibody (Sigma Aldrich, T4026; 1:200) was used as a loading control.

### Construction of mouse cre-lox targeted gene deletion models

Two independently verified [*Exosc10* ^tm1a(KOMP)Wtsi^] targeted embryonic stem (ES) cell clones from the C57BL/6N-A/a background used for this research project (clone IDs EPD0567_5_B04, and EPD0567_5_F03) were generated by the trans-NIH Knock-Out Mouse Project (KOMP) and obtained from the KOMP Repository (www.komp.org). NIH grants to Velocigene at Regeneron (U01HG004085) and the CSD Consortium (U01HG004080) funded the generation of gene-targeted ES cells for 8500 genes that are archived and distributed by the KOMP Repository at UC Davis and CHORI (U42RR024244). The methods used to generate targeted alleles in the C57BL/6N background are described in reference^64^. For more details on the deletion vector, see the KOMP website at www.komp.org/alleles.php#nonconditional-promoter-csd. The ES cell clones were shipped to Institut Clinique de la Souris (ICS, www.ics-mci.fr) and used to generate chimaeric mice that were further bred to achieve germline transmission in five cases (three females and two males). Finally, *Exosc10* ^+/−^ mice were crossed with Rosa26-deleter Flp mice to generate the *Exosc10* ^+/f^ line.

Next, ten *Exosc10* ^+/−^ mice (five males and five females), and seven *Exosc10* ^+/f^ mice (five males and two females) were transferred from the ICS to our local animal facility. Mice generated to study *Exosc10* during embryology will be described elsewhere (Petit *et al*., in preparation). To analyze *Exosc10*’s function during spermatogenesis, we inactivated *Exosc10* in spermatogonia using STOCK Tg(Stra8-icre)1Reb/J (Stra8-cre; The Jackson Laboratory, SN 8208)^37^ and FVB-Tg(Ddx4-cre)1Dcas/J mice (Vasa-cre; The Jackson Laboratory, SN 6954)^36^. Cre-expressing strains were crossed to wild type C57BL/6NRj mice (Janvier, Le Genest Saint Isle, France) at least five times before they were used for experiments. Briefly, Stra8*-*cre female mice were crossed with *Exosc10* ^+/−^ male mice to generate a Stra8-cre; *Exosc10* ^+/−^ mouse line. Females were then mated with *Exosc10* ^f/f^ males to generate Stra8-cre; *Exosc10* ^f/−^ males (*Exosc10* ^cKO(Stra8)^). *Exosc10* ^+/f^ mice were used as controls. In parallel, we used this procedure with Vasa-cre mice to generate the Vasa-cre; *Exosc10* ^f/−^ mouse line (*Exosc10* ^cKO(Vasa)^).

Mouse genotyping was performed using DNA extracted from tail tips and a standard PCR assay. The primer set Ef/Kr/Ef2/Er3 was used to discriminate *Exosc10* ^+/−^, *Exosc10* ^+/f^ and *Exosc10* ^f/−^ genotypes. Since ectopic recombination events can occur when Stra8-cre and Vasa-cre mice are crossed with *Exosc10* floxed mice, we used the primer set Ef/L3r/Ef2/Er3 to detect deletion of the floxed allele by cre recombinase in genomic DNA prepared from tail samples. Oligonucleotide primer sequences are shown in Table 2. Mice used for the experiments were genotyped at weaning and when they were sacrificed, respectively.

**Table 2:** Sequences of oligonucleotides used for PCR genotyping.

Name	DNA Sequence
Ef	5′-GAT GGA GCG AGC AAG CTT CTG-3′
Kr	5′-CCA ACA GCT TCC CCA CAA CGG-3′
Ef2	5′-TCT AAG CCT GAC AGC ATG AAT TGA ACC-3′
Er3	5′-GTG GTG TAG ACT TGT GAT ACT GC-3′
L3r	5′-GCA GGT CTC CAC TGA CAC TG-3′
CREstra8-F	5′-GTG CAA GCT GAA CAA CAG GA-3′
CREstra8-R	5′-AGG GAC ACA GCA TTG GAG TC-3′
CREvasa-F	5′-CAC GTG CAG CCG TTT AAG CCG CGT-3′
CREvasa-R	5′-TTC CCA TTC TAA ACA ACA CCC TGA A-3′

### Fertility assays

Two-months-old control (*Exosc10* ^+/f^) or mutant (*Exosc10* ^cKO(Stra8)^) males were mated with two 8-week-old C57BL/6NRj female mice (Janvier, Le Genest Saint Isle, France). Female mice were replaced by new young females when they reached the age of six months. We recorded each litter date and size to calculate the fertility rate. The assay was terminated when the male reached the age of 12 months.

### Epididymal sperm count

Adult mice were euthanized and the complete epididymis was dissected, weighed, snap frozen in liquid nitrogen and stored at −80 °C. Each epididymis was cut into several pieces in 1 ml 0.15 M NaCl containing 0.05% Triton X-100 (Sigma), and processed using a Polytron PT 10/35 disperser (Kinematica, Luzern, Switzerland). The sperm cells were counted using a Malassez Hemocytometer (Thermo Fisher).

### Tissue collection and histological analysis

Testes and epididymides were collected from male mice at 14 dpp, 8 weeks and 12 months, and each organ was weighed. For mature mice, tissues were fixed in Bouin (right) and the animals were perfused with 4% paraformaldehyde before collecting the left organ. Bouin’s fixative was used for hematoxylin/eosin staining to preserve the testis architecture. Samples were then washed with PBS (pH 7.4), and stored in 70% ethanol. The embedding process was carried out in a Shandon Citadel 2000 Tissue Processor (Thermo Fisher Scientific). Paraffin embedded organs were sectioned at 5 μm on Superfrost slides (Thermo Fisher Scientific). After drying, the slides were rehydrated in the Shandon Varistain 24-3 Automatic Slide Stainer and stained with hematoxylin and eosin (Leica Microsytems).

### Immunohistochemistry analysis

Immunohistochemistry analysis of testicular sections was performed using 4% paraformaldehyde fixed tissues as previously described^65^. Primary antibodies were diluted at 1:50 (GATA1; Santa Cruz Biotechnology sc265), 1:500 (PLZF; R&D Systems AF2944), 1:500 (SYCP3; Abcam ab97672), and 1:200 (STAR; CST 8449 and αSMA; Sigma A2547). Biotinylated polyclonal goat anti-rabbit (Dako, E0432) and anti-mouse (Dako, E0433) antibodies were diluted 1:500. Histological sections were examined using an AxioImager microscope equipped with an AxioCam ICc1 camera and ZEN v.2.3 (Blue edition, Zeiss).

### Statistical analysis

The data were analyzed with Prism 6 (GraphPad) software. The alpha level was set at 0.05. We used the non parametric Mann-Whitney statistical test to compare the testis weight, the epididymis weight, the number of spermatozoa and the percentage of each cell type in control versus mutant mice. The reproductive capacity was analyzed using a *t*-test and the results are presented as the mean value ± SD. The results were considered to be statistically significant when the p-value was <0.05. *p < 0.05, **p < 0.01, ***p < 0.001.

### Data availability

No datasets were generated or analysed during the current study.

## Electronic supplementary material

Supplementary information
