# Supplementary material for: EXOSC10/Rrp6 is post-translationally regulated in male germ cells and controls the onset of spermatogenesis
Source: Sci Rep. 2017 Nov 8;7:15065. doi: 10.1038/s41598-017-14643-y (PMC5678167; doi:10.1038/s41598-017-14643-y)
Supplement: Supplementary file 1 — Supplementary information [file 41598_2017_14643_MOESM1_ESM.pdf]

EXOSC10/Rrp6 is post-translationally regulated in male germ cells and controls the onset of spermatogenesis

Soazik P. Jamin[1,*], Fabrice G. Petit[1], Christine Kervarrec[1], Fatima Smagulova[1], Doris Illner[2,3], Harry Scherthan[2], and Michael Primig[1,*]

[1]Inserm U1085 IRSET, Université de Rennes 1, 35000 Rennes, France

[2]Institut für Radiobiologie der Bundeswehr in Verb. mit der Universität Ulm, 80937 Munich, Germany

[3]present address: PAN-Biotech, 94501 Aidenbach, Germany

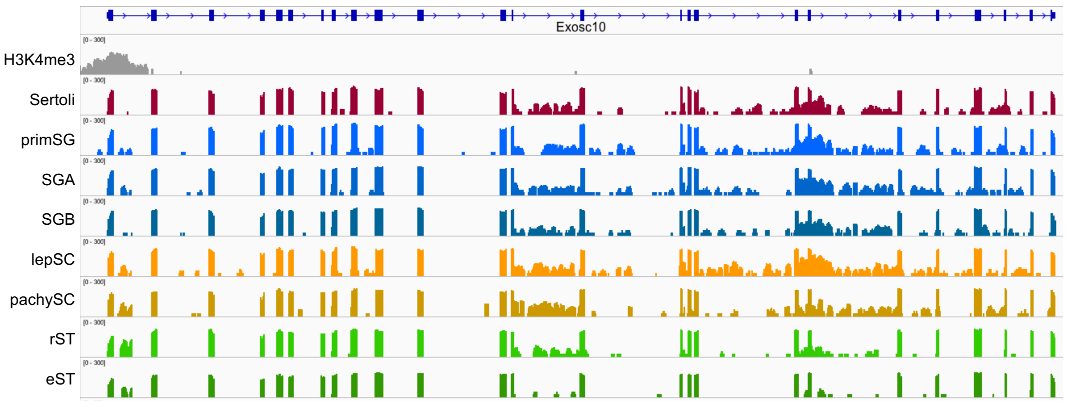

Figure S1. RNA-Seq and epigenetic expression data from testicular cells. A histogram shows the RefSeq annotation of *Exosc10* in blue (top lane); arrowheads point into the direction of transcription. Below are ChIP-Seq data for an epigenetic mark associated with gene activation (histone 3, lysine 4 trimethylation, H3K4me3) in grey, followed by RNA-Seq data obtained with enriched Sertoli cells (Sertoli) in red, primary spermatogonia (primSG), and A and B type spermatogonia (SGA, SGB) in shades of blue, leptotene and pachytene spermatocytes (lepSC, pachySC) in orange and light brown, and round and elongated spermatids (rST, eST) in shades of green.

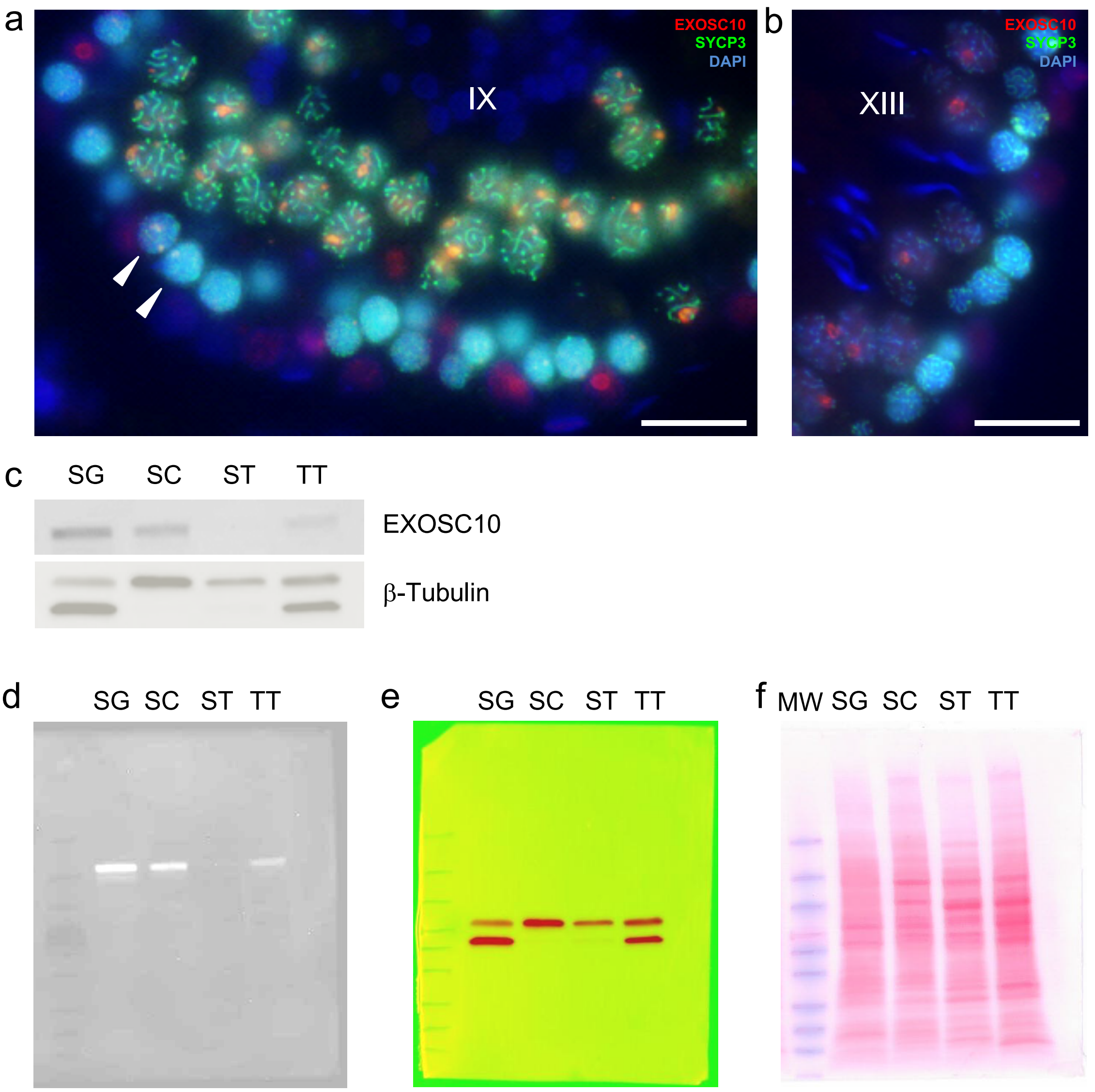

Figure S2. Rat EXOSC10 localization and protein levels in meiotic and post-meiotic germ cells. (a) A triple stained section from a stage IX tubule is shown for EXOSC10 (red), SYCP3 (green) and DNA (blue). White arrowheads point to leptotene spermatocytes. (b) A triple stained section from a stage XIII tubule is shown as in panel a. (c) A Western blot detecting EXOSC10 is given for extracts prepared from spermatogonia (SG), spermatocytes (SC), spermatids (ST) and total testis (TT) samples. β-Tubulin was used as a loading control. (d) Raw image of the membrane assayed with the anti-EXOSC10 antibody. (e) Raw image of the control experiment with the anti-β-Tubulin antibody. Note that the faster migrating band present in spermatogonia (hence, the total testis sample) is likely an isoform as described in Slaughter *et al.*, Biol Reprod 1989. (f) Raw image of the gel stained with Ponceau. Lane one contains molecular weight markers (MW).

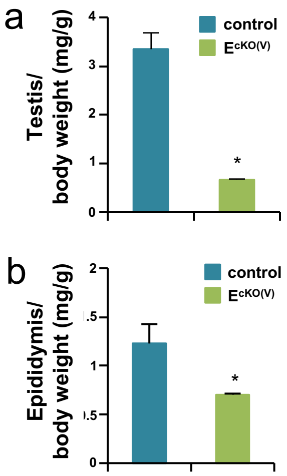

Figure S3. Testis and epididymis weight in Vasa deletion mutants. (a) A color-coded graph plots the weight of testis over body-weight (y-axis) for controls (C in blue) and *Vasa*-cre mutants (*Exosc10*$^{cKO(Vasa)}$ in green) at 2 months (x-axis). (control, n=7; mutant, n=3; p=0.0167 *p<0.05, non parametric Mann-Whitney test). The data are presented as median values ± SD. (b) A color-coded graph plots the weight of epididymides over the body weight. (control, n=6; mutant, n=3; p=0.0238 *p<0.05, non parametric Mann-Whitney test). The data are presented as median values ± SD.
